# Short-lived detection of an introduced vertebrate eDNA signal in a nearshore rocky reef environment

**DOI:** 10.1371/journal.pone.0245314

**Published:** 2021-06-04

**Authors:** Taylor Ely, Paul H. Barber, Lauren Man, Zachary Gold

**Affiliations:** Department of Ecology and Evolutionary Biology, University of California Los Angeles, Los Angeles, California, United States of America; University of Hyogo, JAPAN

## Abstract

Environmental DNA (eDNA) is increasingly used to measure biodiversity of marine ecosystems, yet key aspects of the temporal dynamics of eDNA remain unknown. Of particular interest is *in situ* persistence of eDNA signals in dynamic marine environments, as eDNA degradation rates have predominantly been quantified through mesocosm studies. To determine *in situ* eDNA residence times, we introduced an eDNA signal from a non-native fish into a protected bay of a Southern California rocky reef ecosystem, and then measured changes in both introduced and background eDNA signals across a fixed transect over 96 hours. Foreign eDNA signal was no longer detected only 7.5 hours after introduction, a time substantially shorter than the multi-day persistence times in laboratory studies. Moreover, the foreign eDNA signal spread along the entire 38 m transect within 1.5 hours after introduction, indicating that transport and diffusion play a role in eDNA detectability even in protected low energy marine environments. Similarly, native vertebrate eDNA signals varied greatly over the 96 hours of observation as well as within two additional nearby fixed transects sampled over 120 hours. While community structure did significantly change across time of day and tidal direction, neither accounted for the majority of observed variation. Combined, results show that both foreign and native eDNA signatures can exhibit substantial temporal heterogeneity, even on hourly time scales. Further work exploring eDNA decay from lagrangian perspective and quantifying effects of sample and technical replication are needed to better understand temporal variation of eDNA signatures in nearshore marine environments.

## Introduction

Environmental DNA (eDNA) is increasingly used to investigate biodiversity of marine ecosystems [1]. One source of eDNA is produced when organisms shed genetic material into the environment [2]; by isolating, extracting, and sequencing this eDNA, resident marine species can be identified through metabarcoding [1]. Recent studies demonstrate that eDNA techniques can outperform traditional visual census surveys in species detection [3, 4], particularly for cryptic and rare species [5], while at the same time being noninvasive and cost effective [2]. As such, eDNA represents a promising alternative to traditional biodiversity surveys, which are time and labor intensive, require substantial taxonomic expertise, and can pose significant safety hazards to researchers [2, 6].

Despite the promise of this method, much remains unknown about the dynamics of eDNA in the environment. eDNA degrades in the environment due to a combination of abiotic (e.g. temperature, UV, and pH) and biotic (e.g. microbial activity) processes [7]. Previous studies report that eDNA of marine fishes degrades in a laboratory setting on a scale of 0.5 to 7 days, but usually around 3–4 days [3, 8]. In nature, however, water transport and water mixing affect the persistence and detection of eDNA in the water column, processes that are fundamentally altered in laboratory settings [8].

To date, most eDNA studies examining eDNA transport have focused on single species in freshwater systems characterized by relatively simple flow dynamics such as spawning salmon in streams [e.g. 9, 10]. However, recent work investigating the spatial and temporal variation of *Pseudocaranx dentex* (White trevally) in Maizuru Bay, Japan found that eDNA signatures fell below detection thresholds under just 2 hours after the removal of a foreign eDNA source [11].

This result indicates that *in situ* eDNA signatures can decay much more rapidly than in laboratory experiments, suggesting that the processes generating eDNA signals and processes contributing to signal decay (e.g. degradation, advection, generation, dispersion, and/or diffusion) may be dramatically different in field and laboratory settings. However, it is unclear whether this result is generalizable to all marine ecosystems, including temperate or polar ecosystems where colder water temperatures could slow degradation processes [12].

Understanding temporal variation of eDNA in open marine environments is essential for developing proper eDNA sampling strategies and for interpreting results. For example, although studies report temporal variation in eDNA signatures [13], only a single study explicitly examines short-term temporal variation in eDNA signatures of an entire marine vertebrate community [14]. Kelly et al. [14] found that tides did not have a strong or consistent effect on community composition, but that temperature and salinity did have a significant effect, suggesting that the movement of water masses—rather than tides alone—has the strongest effect on eDNA signatures in an intertidal ecosystem.

Transport of eDNA in marine environments may not strongly impact local eDNA signatures if the eDNA only persists for a few hours, producing a highly localized signal. For example, working in the highly protected waters of Lovers Cove (a northeast facing beach on the west coast of California) Port et al. [15] found that marine vertebrate communities differed on a 60–100 m scale, indicating very limited transport of eDNA, rapid eDNA degradation rates or both [16]. More recent work building off this study in more exposed, dynamic marine environments found that eDNA signatures of marine metazoan and fish communities displayed spatial variation on the scale of hundreds of meters to a few kilometers [17, 18].

Temporal variation of eDNA in marine ecosystems is becoming increasingly important to understand, as eDNA is being viewed as a potential alternative to traditional visual survey methods used in marine ecosystems monitoring [2, 5]. While eDNA has fewer logistical difficulties compared to visual surveys [19], the primary advantage is eDNA’s ability to efficiently detect larger numbers of taxa [3, 4, 15]. For example, at least 178 fish species inhabit Southern California kelp forest [20]. However, the monitoring protocol by Reef Check California only monitors 33 of these species in their visual surveys [21]. Although paired comparisons show that eDNA almost doubled the number of species observed visually [22], it is unclear how stable these community eDNA signatures may be, and how current eDNA sampling protocols reliably capture temporal variation in marine vertebrate communities.

To better understand temporal variation in eDNA signatures, this study investigates the *in situ* persistence of eDNA in a nearshore rocky reef habitat using a Eularian sampling regime. First, we examine the persistence of a point source foreign eDNA signature after introduction along a fixed transect. Second, we investigate how natural eDNA signatures along this transect, as well as two other nearby fixed transects, fluctuated over the time and tide on a short-term scale. Combined, this approach will provide insights into the dissipation of eDNA signatures over time.

## Methods

### Sample collection and filtering

To test for the persistence of eDNA *in situ*, we created a foreign eDNA signature by homogenizing 414 grams of *Ctenopharyngodon idella* (grass carp) muscle tissue in 1L of MilliQ water (EMDMillipore, Burlington, MA) in a blender at high speed for 60 seconds. Tissue was used instead of PCR product because of recent evidence that eDNA derives from whole cells rather that freely associated DNA [8, 22]. Due to the potential for seafood mislabeling [23], we DNA barcoded the tissue sample to verify the species.

**Fig 1:**
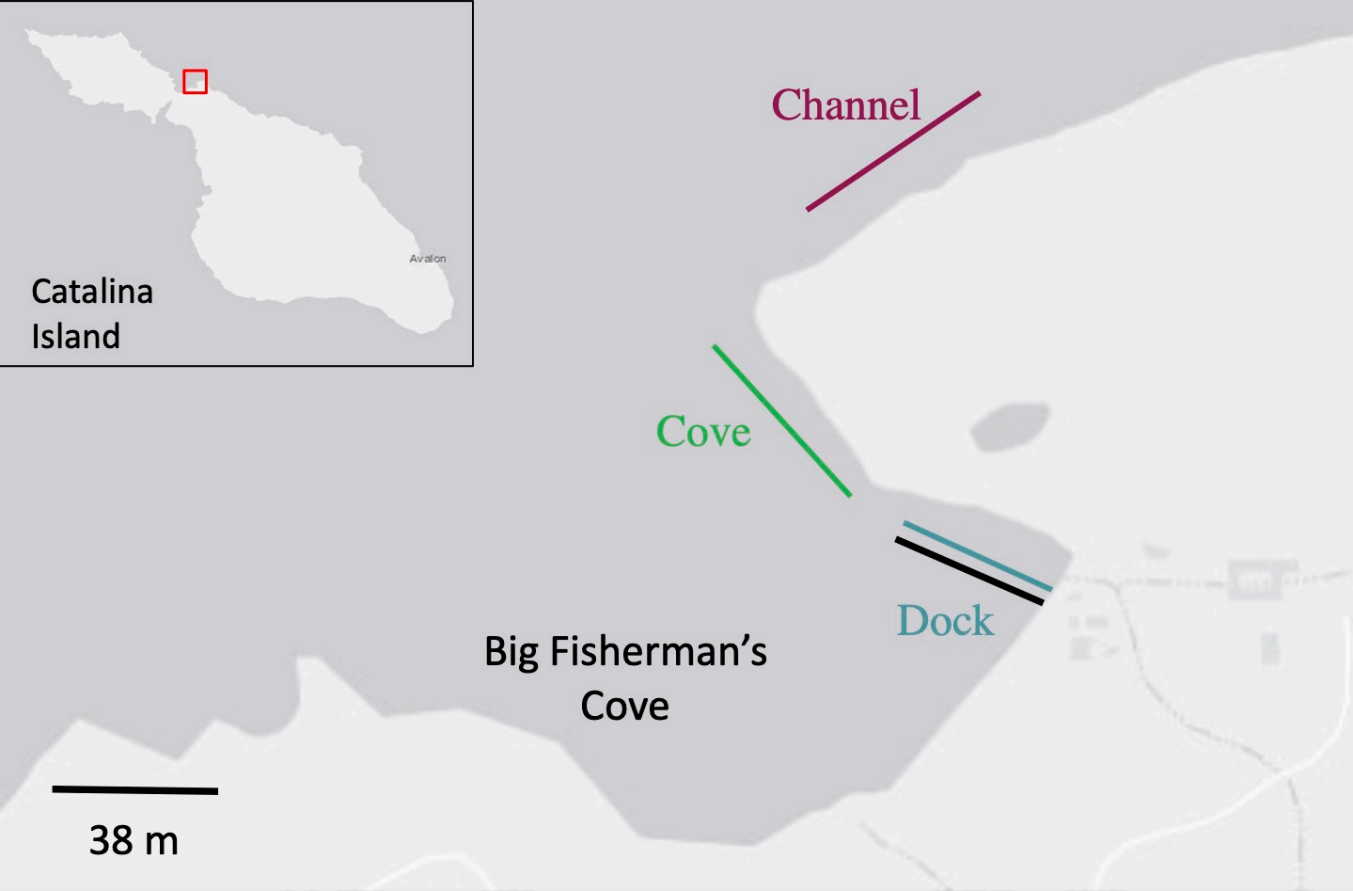
Map of transect locations. The location of Big Fisherman’s Cove on Catalina island and the location of the transects within the cove are displayed. The scale for the cove is in the bottom left corner. Land is represented in white and water in grey. Map services and data available from U.S. Geological Survey, National Geospatial Program.

We conducted fieldwork at the USC Wrigley Marine Science Center on Catalina Island, California, located in Big Fisherman’s Cove (33°26’42.43”N, 118°29’4.05”W). This field station sits within a protected bay within the island wake, experiencing minimal currents and no waves [24]. We followed the methods of previous marine eDNA studies, sampling along fixed transects for biological replicates [13, 25, 26]. In total, we conducted three transects in and around this cove: 1) along the Wrigley Marine Science Center dock (herein dock), 2) starting at the dock and continuing seaward (herein cove), and 3) along the front of the cove along the shipping channel (herein channel) (Fig 1).

For the dock transect, we used the field station’s 38 m long dock to facilitate sampling along a fixed transect without disturbance from SCUBA divers (Fig 1). Replicate A (7.3m depth) was closest to shore, replicate B (8.2 m depth) was 19 m seaward, and replicate C (11.2 m depth) was 19 m further seaward at the end of the dock. Prior to introducing the foreign eDNA signal, we established baseline eDNA signatures by collecting one-liter water samples at each sampling point along dock transect on SCUBA. SCUBA divers then released the total volume of homogenized *C*. *idella* tissue one meter above the sea floor at replicate B on September 6^th^, 2017 at 07:00. We then collected one-liter water samples for eDNA analysis at each of the three dock transect sampling points for a period of 96 hours. For the first 12 hours, we collected samples every 1.5 hours. After this initial period, we sampled only at 24, 48, and 96 hours after the release of the foreign eDNA. Sterile protocols were followed throughout following the guidelines of Goldberg et al. [27].

To eliminate diver related introduction of *C*. *idella* during sample collections, all samples along the dock transect following the introduction of the foreign eDNA signature were taken using a 4-liter Niskin bottle hand-lowered to 1 m above the sea floor. At the surface, we transferred 1 liter of seawater from the Niskin bottle into a sterile 1000 mL kangaroo gravity feeding bag (Covidien, Dublin, Ireland, Product Number 8884702500). This bag was immediately placed in a cooler on ice packs (-20˚C) and transported to the lab, less than 300 m away, for filtration. To ensure that no DNA carried over between sampling events, we cleaned the Niskin bottle between sample collections by rinsing the bottle with surface water above each sampling point for 30 seconds [28]. We also processed a field blank which consisted of one liter of nuclease-free water placed inside the Niskin bottle previously rinsed with locally sourced tap water for 30 seconds. This water was then transferred to the gravity feeding bag and treated identically to all samples.

For the cove and channel transects, we sampled at fixed points of 0, 20, 40, 90, and 140 m (replicate A/B/C/D/E respectively). The depth of points along both transects were 7.3 m ± 1 m. The cove transect started inshore from the end of the dock in Big Fisherman’s Cove and extended seaward towards the entrance of the cove (Fig 1). The channel transect started 160 m NW from the end of the cove transect and continued paralleling the western coastline of the cove entrance (Fig 1). We repeated sampling along both transects at 6, 12, 24, 48, and 120 hours after the initial sampling events on July 24^th^, 2016 at 07:00. While the channel transect was sampled at all replicate and time points, two samples were lost due to laboratory error (replicates A and D of time point 120 hr). At each transect point, we collected a one-liter water sample on SCUBA, one meter above the sea floor, using sterile 1000 mL kangaroo gravity feeding bag (Covidien, Dublin, Ireland, Product Number 8884702500). Upon surfacing, we immediately placed samples in a cooler on ice packs (-20˚C) and transported to the lab, less than 300 m away, for filtration. We processed two field blanks for the cove and channel transects which consisted of one liter of nuclease-free water placed inside the gravity feeding bag. All field blank samples were filtered and processed identically to field samples.

To filter eDNA from the water samples from all collection events, we fitted sterile 0.635 cm diameter Nalgene tubing with a luer-lock adapter to the pouches and connected the sample to a 0.22 μm diameter PVDF Sterivex filter unit (EMDMillipore, SVGPL10RC) [29]. We then hung the bags and attached filters in the lab, allowing samples to gravity filter the total one-liter samples or for a maximum of 40 minutes (S1 Table). Previous filtration efforts demonstrated that if water had not filtered by 40 minutes the filters were clogged and would not allow for additional sample to pass through (data not shown). Following eDNA filtration, we stored filters at -20˚C and transported the filters to UCLA for molecular laboratory work.

### DNA extraction

We extracted DNA from the Sterivex filters using a Qiagen DNeasy Blood and Tissue Kit (Qiagen Inc., Germantown, MD) following protocols from Spens et al. [30] with the following modifications. We added 80 μL of proteinase K and 720 μL ATL buffer from the kit directly into the Sterivex filter before sealing both ends of the filter. We then placed the filters in a rotating incubator overnight at 56°C. Following incubation, we removed the liquid from each Sterivex filter using a sterile 3 mL syringe and transferred the solution into 1.5 mL tubes. We then added equal parts AL buffer and 0˚C ethanol to an equal volume of extracted liquid. The eDNA sample was then extracted with the Qiagen DNeasy Blood and Tissue Kit without any further modifications to the manufacturer’s protocol.

### PCR amplification and DNA sequencing

We amplified the *12S* region of mitochondrial DNA using MiFish Universal Teleost specific primers modified with Illumina Nextera adapter sequences (MiFish-U, S2 Table) [29, 31]. This primer set primarily targets teleost fish but also can detect a wide variety of other vertebrates, including marine mammals and birds [31]. PCR reaction volume was 25 μL and included 12.5 μL Qiagen 2x Multiplex Master Mix, 2.5 μL MiFish-U-F (2 mM), 2.5 μL MiFish-U-R (2 mM), 6.5 μL nuclease-free water, and 1 μL DNA extraction. PCR thermocycling employed a touchdown profile with an initial denaturation at 95°C for 15 min to activate the DNA polymerase followed by 13 cycles with a denaturation step at 94°C for 30 sec, an annealing step with temperature starting at 69.5°C for 30 sec (temperature was decreased by 1.5°C every cycle until 50°C was reached), and an extension step at 72°C for 1 min (S3 Table). Thirty-five additional cycles were then carried out at an annealing temperature of 50°C using the same denaturation and extension steps above, followed by a final extension at 72°C for 10 min (S3 Table). All PCR experiments included negative controls. We then confirmed successful PCR amplification through gel electrophoresis on 2% agarose gels.

To prepare the sequencing library, we first pooled 5 μL from each of the 3 PCR technical replicates. We then purified these PCR products, removing strands less than 100 bp long, using Sera-Mag (Sigma-Aldrich) bead protocol and eluted the purified product in 40 μL nuclease-free water [32]. We attached Illumina Nextera indexing primers to purified products through an indexing PCR [33]. The PCR reaction volume per sample was 25 μL and comprised of 12.5 μL Kapa HiFi Hot Start Ready Mix, 0.625 μL Primer i7, 0.625 μL Primer i5, 6.25 μL nuclease-free water, and 5 μL template (~5 ng). The thermal cycle profile started with 95°C for 5 minutes followed by 5 cycles of denaturing at 98°C for 20 seconds, annealing at 56°C for 30 seconds, and extension at 72°C for 3 minutes. Thermal cycling concluded with a final extension of 72°C for 5 minutes. These indexed samples were again purified to remove strands less than 100 bp long using Sera-Mag beads as described above. DNA concentrations were quantified using the BR Assay Kit (Thermofisher Scientific, Waltham, MA, USA) on a Victor3 plate reader (Perkin Elmer, Waltham, MA, USA). We then generated the final library by pooling equal concentrations of DNA from all indexed samples. Samples from 2016 and 2017 were sequenced separately. The library containing dock transect samples was sequenced at UC Berkeley’s QB3 Genomics. The library containing cove and channel transect samples was sequenced at the Technology Center for Genomics & Bioinformatics (University of California–Los Angeles, CA, USA). Both libraries were sequenced in an Illumina MiSeq Paired end 300x2 sequencing run.

### Bioinformatics

We analyzed the resulting sequences using the *Anacapa Toolkit* (version: 1) [29] identifying the number of reads of *C*. *idella* and amplicon sequence variants (ASVs) from the native vertebrate communities. We used the standard *Anacapa Toolkit* parameters with the *CRUX*-generated *12S* reference library as described in Curd et al. [29] with the addition of 757 barcodes of California fish species [34]. Taxonomic assignment was determined with a Bayesian confidence cutoff score of 60 [35].

We employed an established decontamination workflow following the index hopping removal and low read count thresholds using the methods of Kelly et al. [14]. We then normalized our data using the eDNA index metric, the inversed order of operations of a Wisconsin double-standardization, following the methods Kelly et al. [36]. eDNA indexes were calculated separately for 2016 and 2017 data since they were sequenced separately. This metric assumes that PCR biases originate from template-primer interactions which remain constant across eDNA samples and thus allow us to infer relative abundance changes of a single taxa between samples [36].

### Statistical analysis

To examine degradation of the introduced eDNA, we plotted the index of *C*. *idella* reads at each time point across the first 24 hours of sampling using R (version 3.6.1) [37]. Unlike laboratory studies, we chose not to fit an exponential model to the data as the *C*. *idella* reads did not decrease in a consistent pattern [3, 8]. For comparisons of native vertebrate communities over time, we first visualized which taxa were present in 1) the dock transect in each replicate (A/B/C, N = 3) and time point (0–96 hr, N = 12), in 2) the cove transect in each replicate (A/B/C/D/E, N = 5) and time point (0–120 hr, N = 6), and in 3) the channel transect in each replicate (A/B/C/D/E, N = 5) and time point (0–120 hr, N = 6) by generating heat maps using the R package *phyloseq* (version 1.28.0) [38]. We generated two separate heat maps, one using all taxa detected and a second using only a subset of key ecological indicator species monitored by one or more of local kelp forest monitoring programs (National Park Service Kelp Forest Monitoring Program (KFM), Partnership for Interdisciplinary Studies of Coastal Oceans (PISCO), and Reef Check) (S4 Table).

To determine the number of eDNA samples needed to capture subsets of species diversity, we calculated species accumulation curves for each transect using R package *iNext* (version 2.0.20) [39]. For each transect, we did this to determine how many samples are required to recover 1) all species detected by eDNA over all samples and 2) all species detected by eDNA pooled by replicates over all time points.

Lastly, to test the underlying factors shaping temporal variation in native eDNA community signatures, we investigated how species communities in each transect changed in response to two variables: direction of tide (incoming/outgoing/peak, N = 3) and time point (0–96 hr, N = 12 or 0–120 hr, N = 6). For each transect, we conducted a PERMANOVA test on the Bray-Curtis dissimilarities calculated between each sample to determine the effect of each variable on community composition using the *R* package *vegan* (version 2.5–6) [40]. We chose Bray-Curtis dissimilarities over Jaccard dissimilarities following the methods of Kelly et al. [36] given that eDNA index enables us to infer changes in relative abundance between taxa. We created all graphs and performed all calculations using R (version 3.6.1) [37] with *phyloseq* (version 1.28.0) [38], *Ranacapa* (version 0.1.0) [41], *treemapify* (version 2.5.3) [42], *iNext* (version 2.0.20) [39] and *vegan* (version 2.5–6) [40] packages.

## Results

### Sequencing

For the dock transect, we generated 6,613,832 sequence reads from 36 samples and 5 controls. After decontamination, the number of reads per sample ranged from 54,681–555,952 (mean ± sd = 175,241 ± 11,480 reads/sample), excluding controls, recovering 755 ASVs that were assigned to a total of 99 taxa, 82 to species level. For the cove and channel transects, we generated 6,726,193 reads from 58 samples and 5 controls. After decontamination steps, the number of reads per sample ranged from 14,999–186,248 (mean ± sd = 93,648 ± 40,259 reads/sample), excluding controls, recovering 1,273 ASVs that were assigned to a total of 89 taxa, 81 to species level.

### Temporal variation in foreign eDNA signatures

**Fig 2:**
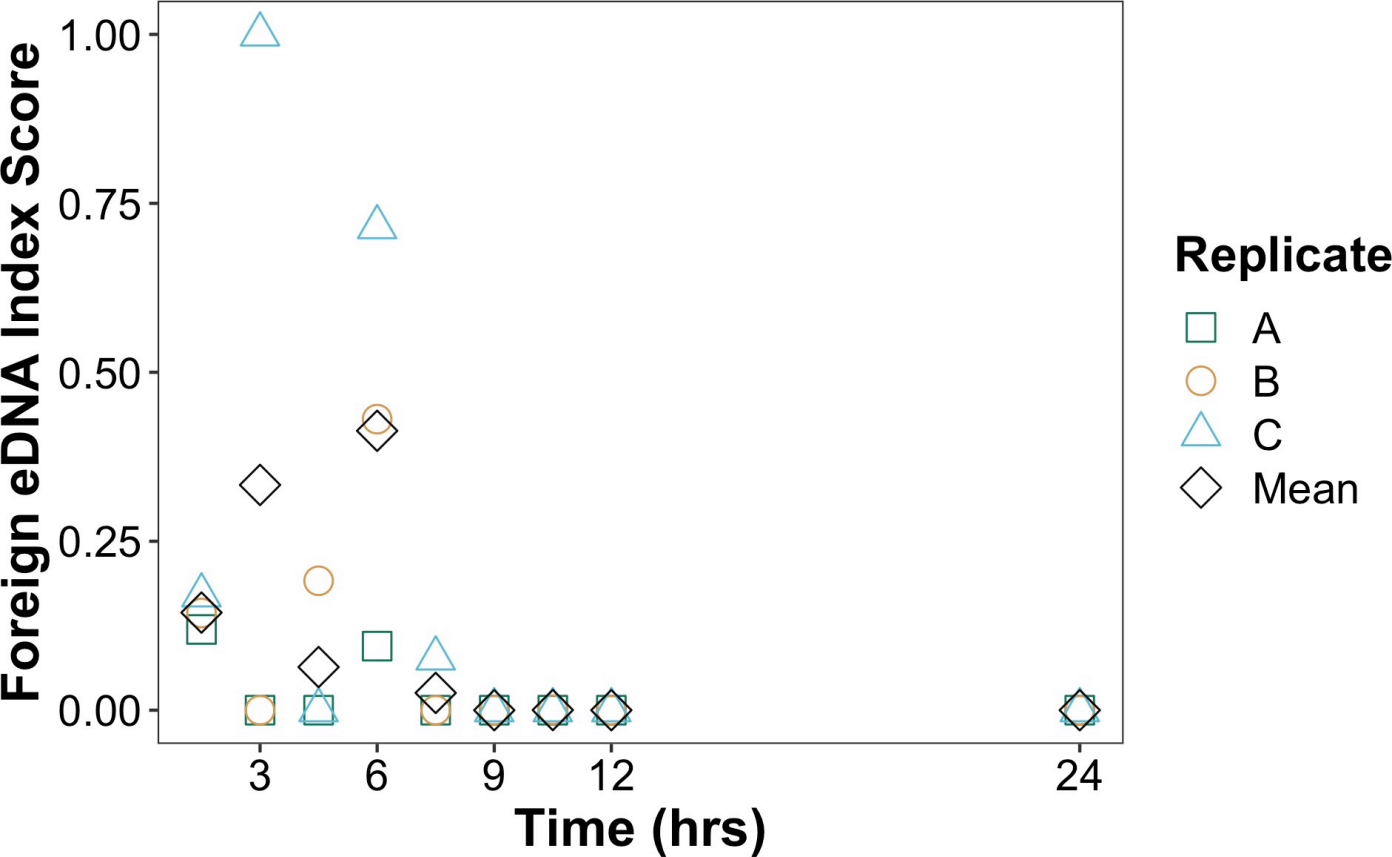
Detection of foreign eDNA over time. Plot of eDNA index for *C*. *idella* detected over time per replicate for the first 24 hours at dock replicates A (green squares), B (orange circles), C (blue triangles), and the mean eDNA index across the transect (black diamonds).

Results showed no *C*. *idella* eDNA in any samples prior to introduction of the tissue homogenate. *C*. *idella* eDNA was detected at all three sites at similarly low detection levels (eDNA index scores = 0.025–0.130) 1.5 hrs after release. eDNA index scores then decreased over time in an inconsistent fashion. The strongest eDNA signature was detected 3 hrs after release, but only in dock replicate C (eDNA index score = 1). No foreign eDNA was detected in replicates A and C at 4.5 hrs, but it was detected again in all replicates at 6 hrs, with replicate C at 6 hrs having the second highest *C*. *idella* eDNA index score over the entire transect (eDNA index score = 0.715). By 7.5 hrs, foreign eDNA was no longer detected (Fig 2).

### Temporal variation in native eDNA signatures

**Fig 3:**
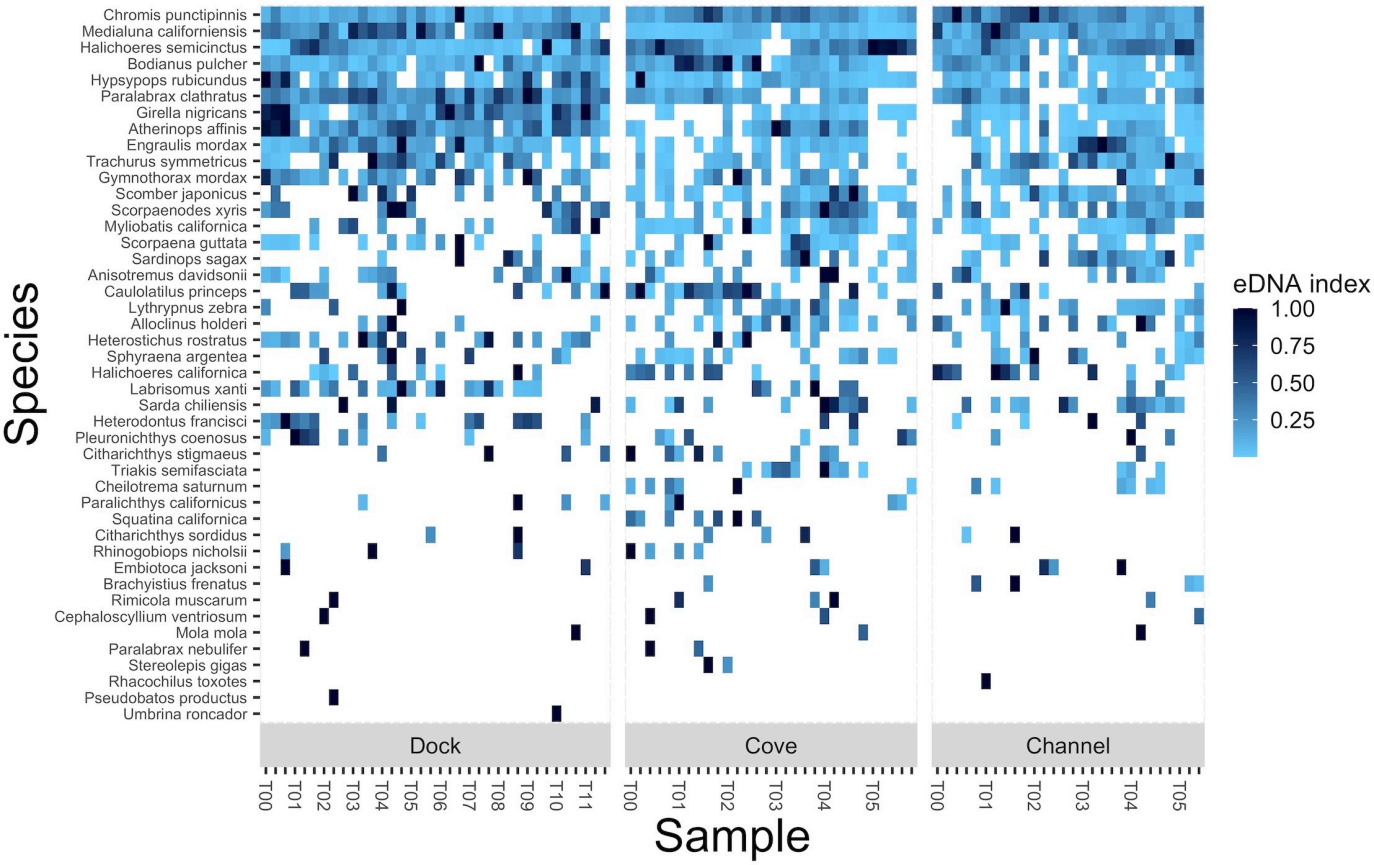
Heat map of species monitored by KFM, PISCO, and reef check detected over time. Heat map showing strength of the eDNA index for species monitored by KFM, PISCO, and Reef Check observed separated by transect: dock, cove, and channel. Darker blue indicates higher index values. White indicates the species was not detected. Samples are ordered by time and then by replicate (A to E) with only the first sample per time point labeled. Species are ordered by decreasing total detections.

Across all dock transect samples, a total of 99 taxa were present in at least one sample across the 3 replicates and 12 sampling times, spanning 4 classes, 29 orders, 55 families, 79 genera, and 82 species (S4 Table). Across all cove transect samples, a total of 76 taxa were present in at least one sample across the 5 replicates and 6 sampling times, spanning 4 classes, 26 orders, 48 families, 67 genera, and 70 species (S4 Table). Across all channel transect samples, a total of 74 taxa were present in at least one sample across the 3 replicates and 12 sampling times, spanning 4 classes, 24 orders, 44 families, 65 genera, and 67 species (S4 Table). Only one species, *Chromis punctipinnis*, was detected in every sample across all three transects (S1A and S2 Figs). Another species, *Medialuna californiensis*, was detected in all channel transect samples but not in all dock or cove transect samples (S1 Fig). Similarly, *Paralabrax clathratus* was detected in all dock samples but not in all cove or channel transects (S1 Fig). The remaining taxa detected exhibited heterogeneous patterns and were absent from one or more sampling points and times; this pattern was also observed for the subset of key ecological indicator species monitored by the KFM, PISCO, and Reef Check (Fig 3 and S2 Fig). We note that the presence of a spike in foreign eDNA in dock transect samples did not reduce the detection of native taxa. The mean number of taxa detected in the dock transect samples when the foreign eDNA signature was present was 21 species (σ = 6) and the mean number of taxa detected in dock transect samples when the foreign eDNA signature was absent was 20 species (σ = 5).

**Fig 4:**
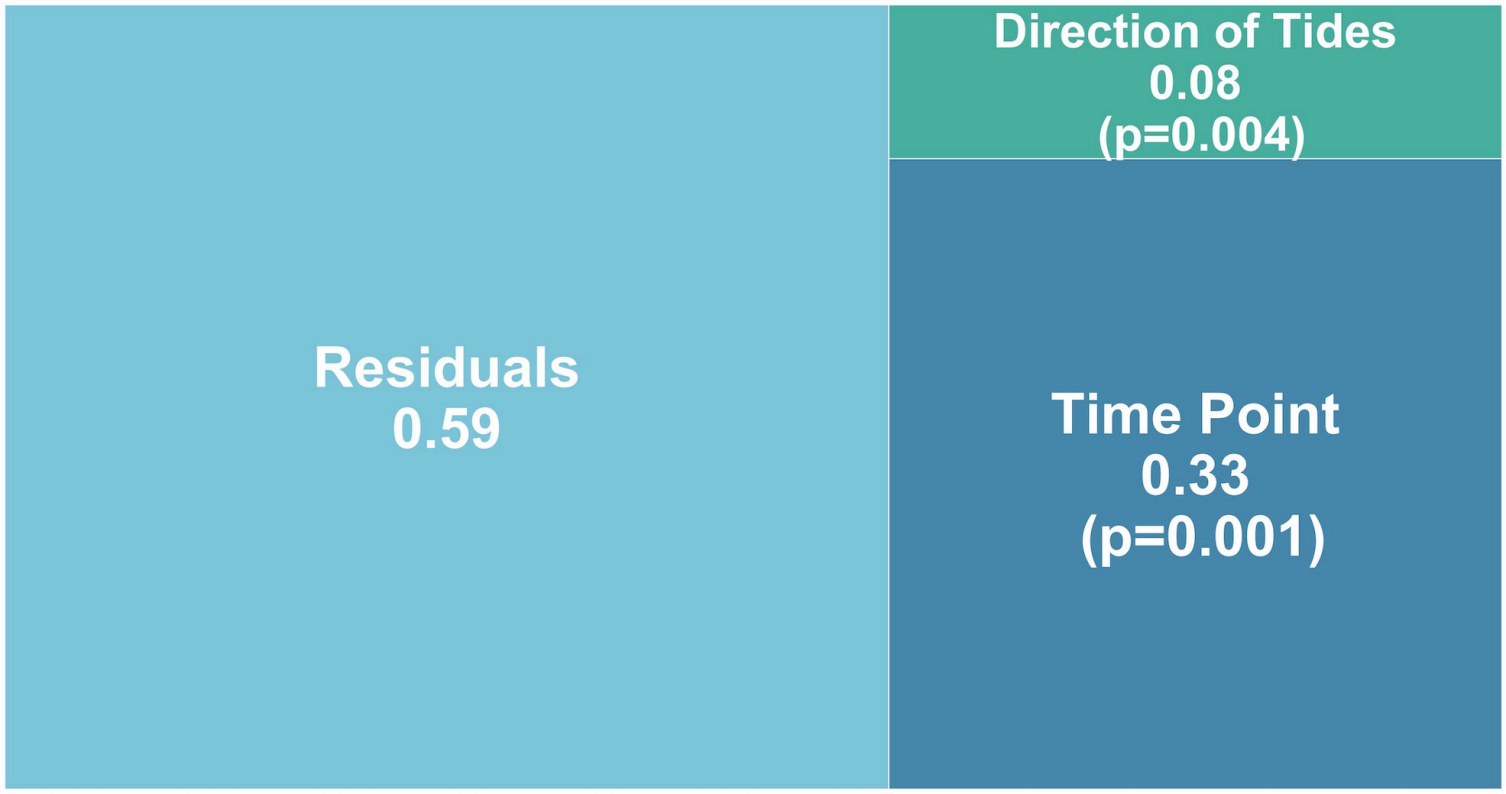
Apportioned variance plot for dock transect from a PERMANOVA with Bray-Curtis dissimilarities. P-values are stated for each factor. The two processes examined are direction of tide (incoming/outgoing/peak) and time point (0–96 hrs).

Time point accounted for the largest portion of variation in vertebrate assemblages in both transect dock and channel transects (PERMANOVA; R^2^ = 33% and 20% respectively) (Fig 4 and S3B Fig). The next most important sources of variation were direction of tide (PERMANOVA; R^2^ = 8% and 14% respectively) (Fig 4 and S3B Fig). The remaining 59% and 66%, respectively, of variation was unaccounted for (Fig 4 and S3B Fig). Time point and direction of tide were all significantly correlated with Bray-Curtis community structure for dock transect (PERMANOVA; F_9_ = 1.4602 and F_2_ = 1.5678 respectively; p = 0.001 and 0.004 respectively) (Fig 4) and for channel transect (PERMANOVA; F_3_ = 2.3042 and F_2_ = 2.2937 respectively; p = 0.001 and 0.001 respectively) (S3B Fig). In the cove transect, direction of tides accounted for the most variation in vertebrate assemblages (PERMANOVA; R^2^ = 19%, F_2_ = 3.8365, p = 0.001) (S3A Fig). Time point also explained a similar degree of variation (PERMANOVA; R^2^ = 18%, F_3_ = 2.3274, p = 0.001) (S3A Fig). The remaining 62% was unaccounted for (S3A Fig). Assumptions for all above PERMANOVA tests were met for all factors (betadisper > 0.05, S5 Table). NMDS ordination plots showed very weak clustering with no discernible effect of time or tide for both dock and channel transects (Stress > 0.25; S4 and S5 Figs respectively). Although the NMDS ordination plot for cove transect showed some clustering by time and tide, stress was weak (Stress > 0.25; S4 and S5 Figs).

**Fig 5:**
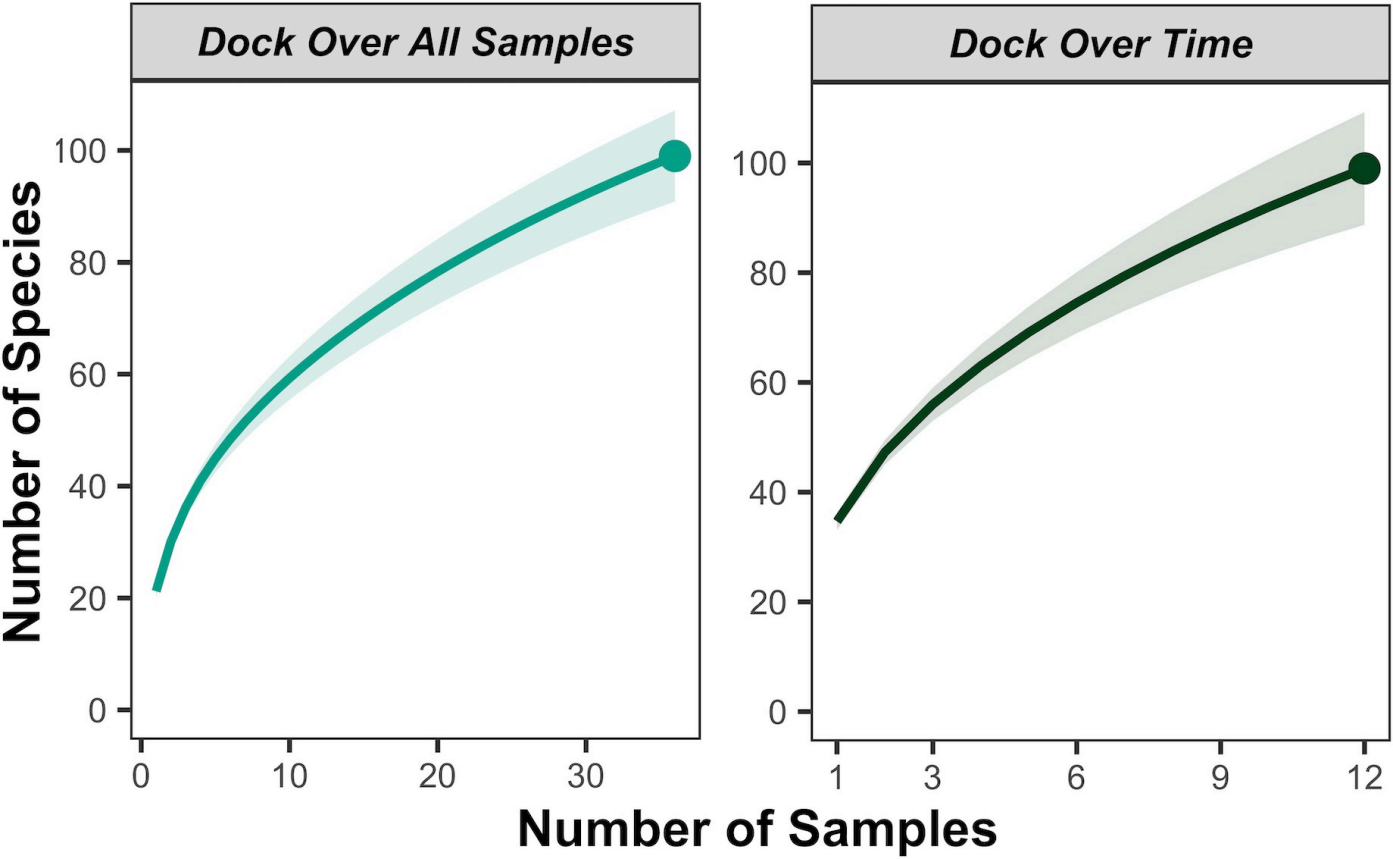
eDNA metabarcoding species accumulation curves for the dock transect. Species accumulation curves quantify how many species on average are detected with increasing number of samples taken. The left graph is a species accumulation curve for total marine vertebrate diversity in each replicate over all samples of dock transect. The right graph is a species accumulation curve for total marine vertebrate diversity along the transect over all time points of dock transect.

Species accumulation curves for dock, cove, and channel transects showed that species richness approached saturation, with a species capture estimate of 95.0%, 97.6%, and 96.2% respectively, across all samples per transect (Fig 5, S6A and S6B Fig). Species capture estimates for three random samples, the typical eDNA sampling protocol, for the dock, cove, and channel transects were 77.4%, 78.1%, and 78.9%, respectively, when focusing only on ecological indicator species monitored by KFM, Reef Check, and PISCO. Across time points, species capture estimates for dock, cove, and channel transects were 90.7%, 94.0%, and 88.1%, respectively (Fig 5, S6A and S6B Fig).

## Discussion

### Temporal variation in foreign eDNA signatures

In contrast to aquaria and mesocosm studies showing eDNA persisting for multiple days [3, 8], this study demonstrates that *in situ* eDNA signals can be short lived, falling below detection thresholds in only 7.5 hrs. These results are highly similar to results from temperate waters in Japan [11], indicating that eDNA signals can dissipate rapidly in temperate marine environments through degradation and/or advection, even though cooler waters may experience slower degradation rates [12]. The ephemeral nature of eDNA is further supported by the variation in the detection of local fish communities sampled repeatedly at the exact same locations over a period of 4–5 days. Combined, these results suggest that persistence times of eDNA in dynamic marine environments is likely much shorter than currently believed.

While degradation certainly contributed to the dissipation of the introduced eDNA signal over time, advection was also likely a contributing factor. The detection of our introduced eDNA at both sites 19 meters away, only 1.5 hours after release, indicates that the eDNA was moving in both directions at a rate of at least 12 m/hr. While not particularly fast, this rate was fast enough to impact eDNA detection. Previous studies in highly dynamic marine environments show that eDNA can be transported tens of kilometers [43]. However, our study occurred in a protected bay with relatively limited water movement, so advection was not expected to play a major role in eDNA signal detection. This surprising result indicates that eDNA transport may play a significant role in the ability to detect eDNA signals, even in relatively protected marine ecosystems, and may explain the high variability in species detection across time at our fixed sampling points. Thus, accounting for fine-scale physical oceanography and transport processes may be critical when designing eDNA sampling regimes.

### Temporal variation in native eDNA signatures

As with the introduced eDNA signature, the detection of the native vertebrate communities was transient and highly variable. The majority of taxa recovered by eDNA would be classified as “resident” taxa, such as kelp bass (*P*. *clathratus*) and garibaldi (*Hypsypops rubicundus*). However, only one species, the damselfish *C*. *punctipinnis*, was observed in all samples. A small number of common kelp forest fishes were seen in the majority (>85%) of samples (e.g. *Halichoeres semicinctus*, *M*. *californiensis*, *Bodianus pulcher*), but the vast majority of taxa were only detected intermittently in eDNA samples (S1A and S2 Figs). Intermittent detection is expected for the highly mobile/migratory species observed in our samples such as ocean sunfish (*Mola mola*) or Risso’s dolphins (*Grampus griseus*). However, it is surprising that species with demonstrated high site fidelity (e.g. *Halichoeres californica*) [44] have intermittent eDNA signatures at fixed sampling locations.

Interestingly, time of sampling explained the largest amount of variation recovered by eDNA for both dock and channel transect communities. The most likely reason for this result is the autocorrelation between time of day and tides over the experiment. However, for the dock and channel transects only 8–14% of the variation in eDNA signatures was explained by the direction of the tide, indicating that tidal advection was not a major driver of temporal variation in eDNA signatures at these sites. Interestingly, at the cove transect there was a higher degree of variation explained by tide (19%). Even so, ≥59% of variation remained unexplained across all three transects. Other processes related to time that may cause variation in community structure could include fish behavior and activity patterns. For example, more fish movement could lead to more sloughing of eDNA, or predation on fish like anchovy, sardines, or silversides could result in spikes of eDNA as injured fish release blood or other tissues into the water. Alternatively, given that DNA is broken down by UV light, eDNA degradation could be faster during times of high solar irradiance, however there was no obvious temporal pattern observed in the presence/absence of species in this study.

Although the majority of the detected species were resident demersal fish species, there was substantial variation in the community composition recovered across temporal eDNA sampling. The highly localized nature of this eDNA signal suggests that eDNA applications that require robust, reliable, and repeatable data on community composition (e.g. marine protected area monitoring) will require multiple replicates to detect all species within a given marine environment. These findings are unsurprising given the nature of species distributions in ecosystems and imperfect sampling methods [45].

### Utility of eDNA in detecting monitored taxa

In total, eDNA recovered broader species diversity than visual survey protocols. Across all samples of a transect, eDNA detected 67–82 species, 35–39 of which are key ecological indicators monitored by KFM [46], Reef Check [21], or PISCO [47]. Interestingly, many of these monitored species observed were only found in a single sample (Fig 3 and S1 Fig), indicating stochasticity in eDNA surveys, akin to that of visual fish surveys. Although we recovered 74 to 99 taxa, this total required 30 to 36 samples over 96 to 120 hours. Typically, eDNA studies use only 3 biological replicates at a single time point [3, 25, 48]. When only 3 random samples were used, eDNA captured an average of 37 total taxa. When only using a single, random 1 L sample, eDNA captured an average of 20 total taxa.

### Other considerations

Perhaps one of the more perplexing results of this study was the high variation in community diversity observed across replicates in our eDNA samples, and the inability to link these patterns to any physical processes that could explain the majority of this variation. Groups overlapped on the Bray-Curtis dissimilarities NMDS ordination plots (S4 and S5 Figs), and there was no clear pattern of prevalence on the heatmaps (Fig 3 and S2 Fig). Furthermore, most variation could not be accounted for by either of the processes (tides and time) analyzed. Other potential sources of variation of community structure observed include PCR bias, fish behavior, and the volume of water sampled.

Kelly et al. [14] reports that PCR replicates and amplification bias accounted for 12–38% of the variance in intertidal communities, suggesting that PCR variation could be a potential significant driver of our observed variation. Similar results were found by Doi et al. [45] and from simulation results by Kelly et al. [36]. Although we performed PCR in triplicate to limit the impacts of PCR bias, those technical replicates were pooled, rather than being sequenced individually. While we cannot directly assess variation among our PCR amplifications, there is no reason to believe that pooling before indexing (our protocol), versus indexing before pooling (Kelly et al. protocol) should make a difference in terms of variation among sequencing, as ultimately the same amount of product from PCR reactions is loaded onto the sequencer. However, a few studies have shown that three PCR replicates fail to eliminate variation due to PCR bias, with PCR bias still resulting in ≥50% variation [49, 50]. Thus, although our study design precludes direct observation of variation across PCR replicates and takes measures to reduce PCR bias, PCR bias likely explains a portion of the 59–66% unaccounted variation in this study.

Another possibility is that physical movement of fish in this environment could drive the movement of eDNA signatures. Katija and Dabiri [51] showed how diel migration of plankton resulted in biogenic mixing of ocean surface waters, and fish can likely do the same [52]. Providing vertical structure and occurring in a marine protected area, the dock at Wrigley Marine Science Center attracts large numbers of schooling fish (*pers*. *obs*.). Thus, although this site is well-protected from currents, biogenic mixing could facilitate the transport of eDNA in multiple directions, and perhaps explain some of the stochasticity in recovered eDNA signals.

Lastly, our sample design of filtering one-liter of water may result is some variation between replicates. The degree of homogeneity of eDNA in the water column is largely unknown. Patchiness in marine fish eDNA has been observed in a tropical ecosystem with < 60% overlap in species composition among aliquots from a water sample [53]. If marine fish eDNA was patchy in this experiment, filtering a larger volume of water may reduce variation among the replicates. However, we chose one liter as our sampling volume since this is the standard sampling volume for marine eDNA studies [14, 15, 17, 19, 26, 48], and passing more than one liter of water through a filter can be difficult in productive coastal ecosystems like kelp forests.

Surprisingly, even when we combined all samples per transect, species accumulation curves suggest that additional sampling is needed to detect all vertebrate biodiversity present. Most eDNA studies sample 1–4 biological replicates taken simultaneously and at most two separate time points unless explicitly observing temporal patterns [3, 17, 18, 54, 55]. Based on the results of this study, such sparse sampling efforts may not yield the most complete picture of local diversity. While our study did find high temporal variation, we also found high variation among replicates from a single time point. Unfortunately, we did not design our study to investigate if many replicates at a single time point can recover the same community diversity as multiple temporal replicates. Further tests are required to determine whether species detection could be maximized while maintaining efficiency by pooling multiple water samples or multiple eDNA extractions, allowing broader sampling without increasing the number of PCR and library preparations that would result in significantly greater lab costs.

Although eDNA was able to capture a broad array of local fish diversity, we note that some taxonomic assignments were made to taxa that are not native to California, but which have closely related taxa in California coastal waters. This phenomenon has been reported previously in Southern California waters [29], and is a function of not accounting for biogeography in closely related taxa with limited genetic variation at the MiFish 12S locus [34]. Improving the accuracy and effectiveness of eDNA metabarcoding could be achieved through expanded barcoding efforts that create more complete and accurate reference databases, as well as using species ranges to inform assignments of closely related species. Importantly, it is critical to archive metabarcoding datasets because bioinformatic pipelines like *Anacapa Toolkit* [29] make it straightforward to rerun legacy datasets as reference databases become more complete.

## Conclusion

While eDNA holds promise to improve the way that we monitor marine biodiversity, much remains to be learned about the dynamics of eDNA in the natural environment. Diffusion and transport of eDNA, not just degradation, impact our ability to detect taxa within the marine environment, resulting in heterogeneity that may not faithfully reconstruct local communities. However, the impacts of these processes can be minimized by increasing the sampling effort, allowing diversity estimates to converge on the most complete reconstruction of local communities. Likewise, sampling efforts can be scaled down if monitoring focuses on a smaller subset of ecological indicator taxa. As marine environments worldwide continue to be impacted by anthropogenic stressors and climate change [56], it will be essential to continue to develop and refine eDNA sampling strategies, allowing this method to achieve its promise as a rapid, reliable, repeatable, and affordable tool for marine ecosystem monitoring [57] in support of marine ecosystem management.

## Supporting information

S1 Fig Detection rates of monitored species. Histogram of the percentage of samples each species was detected in for all species monitored by Reef Check, Partnership for Interdisciplinary Studies of Coastal Oceans (PISCO), or National Park Service (KFM) observed for **(A)** all three transects, **(B)** only dock transect, **(C)** only cove transect, and **(D)** only channel transect.
(TIF)

S2 Fig Heat map of all taxa detected. The heat map is ordered by time point then by replicate (in order of location A to E) and faceted by transect. Darker blue indicates higher eDNA index scores, interpreted as higher relative prevalence. White indicates the taxa was not detected.
(TIF)

S3 Fig Apportioned variance plots from a PERMANOVA with Bray-Curtis dissimilarities for (A) cove transect and (B) channel transect. P-values are stated for each factor. The two processes examined are direction of tide (incoming/outgoing/peak, N = 3) and time point (0–120 hrs, N = 6).
(TIF)

S4 Fig NMDS ordination of community assemblages over time. NMDS ordination plot of community assemblages using all taxa observed with Bray-Curtis dissimilarities faceted by transect. The plot is colored and filled by time point.
(TIF)

S5 Fig NMDS ordination of community assemblages over tides. NMDS ordination plot of community assemblages using all taxa observed with Bray-Curtis dissimilarities faceted by transect. The plot is colored and filled by direction of tide (incoming/outgoing/peak). Shapes also correlate with direction of tide (incoming/outgoing/peak).
(TIF)

S6 Fig Species accumulation curves. Species accumulation curves which indicate how many species on average are detected with increasing number of samples taken for **(A)** cove transect and for **(B)** channel transect. The left graph is a species accumulation curve for total marine vertebrate diversity in each replicate over all samples of each transect. The right graph is a species accumulation curve for total marine vertebrate diversity along the transect over all time points of each transect.
(TIF)

S1 Table Sample volumes filtered. Volume of water in mL filtered through for each sample from the dock transect. All samples from the cove and channel transects filtered all 1000 mL of water.
(XLSX)

S2 Table Primers sequences. Includes the primer name, target species, primer sequence (5’ to 3’), Illumina Nextera index adapter, and target fragment length. Underlined and bolded sequences represent the original MiFish-U primer set.
(XLSX)

S3 Table Touchdown PCR thermal profile. (XLSX)

S4 Table Decontaminated data table. All taxa detected after data was cleaned. Each taxa states if it is a known native, if it is monitored by Reef Check, Partnership for Interdisciplinary Studies of Coastal Oceans (PISCO), or National Park Service (NPS), if it was detected at dock, cove, and/or channel transects, and the number of ASVs assigned to each taxa in 2016 (cove and channel) and 2017 (dock) data. Blank cells indicate “No”.
(XLSX)

S5 Table Betadisper p-values. Betadisper analysis was run for each factor of each transect to ensure assumptions for PERMANOVA were met. All p-values were insignificant (p > 0.05) indicating that assumptions were met.
(XLSX)

## References

[1] Deiner K, Renshaw MA, Li Y, Olds BP, Lodge DM, Pfrender ME. Long-range PCR allows sequencing of mitochondrial genomes from environmental DNA. Methods Ecol Evol. 2017;8(12):1888–98.

[2] Kelly RP, Port JA, Yamahara KM, Martone RG, Lowell N, Thomsen PF, et al. Harnessing DNA to improve environmental management. Science (80-). 2014;344(6191):1455–6. doi: 10.1126/science.1251156 24970068

[3] Thomsen PF, Kielgast J, Iversen LL, Møller PR, Rasmussen M, Willerslev E. Detection of a Diverse Marine Fish Fauna Using Environmental DNA from Seawater Samples. PLoS One. 2012;7(8):1–9. doi: 10.1371/journal.pone.0041732 22952584 PMC3430657

[4] Valentini A, Taberlet P, Miaud C, Civade R, Herder J, Thomsen PF, et al. Next-generation monitoring of aquatic biodiversity using environmental DNA metabarcoding. Mol Ecol. 2016;25(4):929–42. doi: 10.1111/mec.13428 26479867

[5] Bohmann K, Evans A, Gilbert MTP, Carvalho GR, Creer S, Knapp M, et al. Environmental DNA for wildlife biology and biodiversity monitoring. Trends Ecol Evol. 2014;29(6):358–67. doi: 10.1016/j.tree.2014.04.003 24821515

[6] Usseglio P. Quantifying reef fishes: Bias in observational approaches. Ecology of Fishes on Coral Reefs. 2015. 270–273 p.

[7] Barnes MA, Turner CR, Jerde CL, Renshaw MA, Chadderton WL, Lodge DM. Environmental conditions influence eDNA persistence in aquatic systems. Environ Sci Technol. 2014;48(3):1819–27. doi: 10.1021/es404734p 24422450

[8] Sassoubre LM, Yamahara KM, Gardner LD, Block BA, Boehm AB. Quantification of Environmental DNA (eDNA) Shedding and Decay Rates for Three Marine Fish. Environ Sci Technol. 2016;50(19):10456–64. doi: 10.1021/acs.est.6b03114 27580258

[9] Shogren AJ, Tank JL, Andruszkiewicz E, Olds B, Mahon AR, Jerde CL, et al. Controls on eDNA movement in streams: Transport, Retention, and Resuspension. Sci Rep. 2017;7(1):1–11. doi: 10.1038/s41598-016-0028-x 28698557 PMC5506058

[10] Tillotson MD, Kelly RP, Duda JJ, Hoy M, Kralj J, Quinn TP. Concentrations of environmental DNA (eDNA) reflect spawning salmon abundance at fine spatial and temporal scales. Biol Conserv. 2018;220(January):1–11.

[11] Murakami H, Yoon S, Kasai A, Minamoto T, Yamamoto S, Sakata MK, et al. Dispersion and degradation of environmental DNA from caged fish in a marine environment. Fish Sci. 2019;85(2):327–37.

[12] Jo T, Murakami H, Yamamoto S, Masuda R, Minamoto T. Effect of water temperature and fish biomass on environmental DNA shedding, degradation, and size distribution. Ecol Evol. 2019;9(3):1135–46. doi: 10.1002/ece3.4802 30805147 PMC6374661

[13] Kelly RP, O’Donnell JL, Lowell NC, Shelton AO, Samhouri JF, Hennessey SM, et al. Genetic signatures of ecological diversity along an urbanization gradient. PeerJ. 2016;2016(9). doi: 10.7717/peerj.2444 27672503 PMC5028742

[14] Kelly RP, Gallego R, Jacobs-Palme E. The effect of tides on nearshore environmental DNA. PeerJ. 2018;2018(3). doi: 10.7717/peerj.4521 29576982 PMC5863721

[15] Port JA, O’Donnell JL, Romero-Maraccini OC, Leary PR, Litvin SY, Nickols KJ, et al. Assessing vertebrate biodiversity in a kelp forest ecosystem using environmental DNA. Mol Ecol. 2016;25(2):527–41. doi: 10.1111/mec.13481 26586544 PMC4737306

[16] Dell’Anno A, Corinaldesi C. Degradation and turnover of extracellular DNA in marine sediments: Ecological and methodological considerations. Appl Environ Microbiol. 2004;70(7):4384–6. doi: 10.1128/AEM.70.7.4384-4386.2004 15240325 PMC444808

[17] O’Donnell JL, Kelly RP, Shelton AO, Samhouri JF, Lowell NC, Williams GD. Spatial distribution of environmental DNA in a nearshore marine habitat. PeerJ. 2017;2017(2):1–24. 10.7717/peerj.3044 PMC5333549 28265513

[18] Yamamoto S, Masuda R, Sato Y, Sado T, Araki H, Kondoh M, et al. Environmental DNA metabarcoding reveals local fish communities in a species-rich coastal sea. Sci Rep. 2017;7:1–12. doi: 10.1038/s41598-016-0028-x 28079122 PMC5227697

[19] Gold Z, Sprague J, Kushner DJ, Zerecero E, Barber PH. eDNA metabarcoding as a biomonitoring tool for marine protected areas. PLoS ONE. 2021;16(2): e0238557 doi: 10.1371/journal.pone.0238557 33626067 PMC7904164

[20] Kushner D, Rassweiler A, MacLaughlin J, Lafferty K. A multi-decade time series of kelp forest community structure at the California Channel Islands. Ecology. 2013;94(2655).

[21] Freiwald J, Wisniewski C, Wehrenberg M, Shuman C, Dawson C. Reef Check California Instruction Manual: A Guide to Rocky Reef Monitoring, 8th Edition. Reef Check Found. 2015.

[22] Turner CR, Barnes MA, Xu CCY, Jones SE, Jerde CL, Lodge DM. Particle size distribution and optimal capture of aqueous macrobial eDNA. Methods Ecol Evol. 2014;5(7):676–84.

[23] Willette DA, Simmonds SE, Cheng SH, Esteves S, Kane TL, Nuetzel H, et al. Using DNA barcoding to track seafood mislabeling in Los Angeles restaurants. Conserv Biol. 2017;31(5):1076–85. doi: 10.1111/cobi.12888 28075039

[24] Caldeira RMA, Marchesiello P, Nezlin NP, DiGiacomo PM, McWilliams JC. Island wakes in the Southern California Bight. J Geophys Res Ocean. 2005;110(11):1–20.

[25] Nichols PK, Marko PB. Rapid assessment of coral cover from environmental DNA in Hawai’i. Environ DNA. 2019;1(1):40–53.

[26] Nguyen BN, Shen EW, Seemann J, Correa AMS, O’Donnell JL, Altieri AH, et al. Environmental DNA survey captures patterns of fish and invertebrate diversity across a tropical seascape. Sci Rep. 2020;10(1):1–14. doi: 10.1038/s41598-019-56847-4 32317664 PMC7174284

[27] Goldberg CS, Turner CR, Deiner K, Klymus KE, Thomsen PF, Murphy MA, et al. Critical considerations for the application of environmental DNA methods to detect aquatic species. Methods Ecol Evol. 2016;7(11):1299–307.

[28] Thomsen P, Bach S, Sigsgaard E, Moeller P. Monitoring Aquatic Biodiversity in the Gulf using Environmental DNA. Qatar Found Annu Res Conf Proc. 2016.

[29] Curd EE, Gold Z, Kandlikar GS, Gomer J, Ogden M, O’Connell T, et al. Anacapa Toolkit: An environmental DNA toolkit for processing multilocus metabarcode datasets. Methods Ecol Evol. 2019;10(9):1469–75.

[30] Spens J, Evans AR, Halfmaerten D, Knudsen SW, Sengupta ME, Mak SST, et al. Comparison of capture and storage methods for aqueous macrobial eDNA using an optimized extraction protocol: advantage of enclosed filter. Methods Ecol Evol. 2017;8(5):635–45.

[31] Miya M, Sato Y, Fukunaga T, Sado T, Poulsen JY, Sato K, et al. MiFish, a set of universal PCR primers for metabarcoding environmental DNA from fishes: Detection of more than 230 subtropical marine species. R Soc Open Sci. 2015;2(7). doi: 10.1098/rsos.150088 26587265 PMC4632578

[32] Faircloth B, Glenn T. Protocol: preparation of an AMPure XP substitute (AKA Serapure). Web Doc 2014;10:J9MW2F26.

[33] Rohland N, Reich D. Cost-effective, high-throughput DNA sequencing libraries for multiplexed target capture. Genome Res. 2012;22(5):939–46. doi: 10.1101/gr.128124.111 22267522 PMC3337438

[34] Gold Z, Choi E, Kacev D, Frable B, Burton R, Thompson A, et al. FishCARD: Fish 12S California Current Specific Reference Database for Enhanced Metabarcoding Efforts. Authorea. 2020;1–14.

[35] Gao X, Lin H, Revanna K, Dong Q. A Bayesian taxonomic classification method for 16S rRNA gene sequences with improved species-level accuracy. BMC Bioinformatics. 2017;18(1):1–10. doi: 10.1186/s12859-016-1414-x 28486927 PMC5424349

[36] Kelly RP, Shelton AO, Gallego R. Understanding PCR Processes to Draw Meaningful Conclusions from Environmental DNA Studies. Sci Rep. 2019;9(1):1–14. doi: 10.1038/s41598-018-37186-2 31431641 PMC6702206

[37] Team RC. R: A language and environment for statistical computing. 2019.

[38] McMurdie PJ, Holmes S. Phyloseq: An R Package for Reproducible Interactive Analysis and Graphics of Microbiome Census Data. PLoS One. 2013;8(4). 10.1371/journal.pone.0061217 PMC3632530 23630581

[39] Hsieh TC, Ma KH, Chao A. iNEXT: an R package for rarefaction and extrapolation of species diversity (Hill numbers). Methods Ecol Evol. 2016;7(12):1451–6.

[40] Oksanen J, Blanchet FG, Friendly M, Kindt R, Legendre P, McGlinn D, et al. vegan: Community Ecology Package [Internet]. 2019. Available from: https://cran.r-project.org/web/packages/vegan/index.html doi: 10.3389/fmicb.2019.02371 31708882 PMC6824217

[41] Kandlikar GS, Gold ZJ, Cowen MC, Meyer RS, Freise AC, Kraft NJB, et al. Ranacapa: An R package and shiny web app to explore environmental DNA data with exploratory statistics and interactive visualizations [version 1; referees: 1 approved, 2 approved with reservations]. F1000Research. 2018;7(0):1–18. doi: 10.12688/f1000research.16680.1 30613396 PMC6305237

[42] Wilkins D. Treemapify [Internet]. Available from: https://github.com/wilkox/treemapify

[43] Thomas LN, Tandon A, Mahadevan A. Submesoscale processes and dynamics. Ocean Model an Eddying Regime Geophys Monogr Ser. 2008;17–38.

[44] Hartney KB. Site fidelity and homing behaviour of some kelp-bed fishes. J Fish Biol. 1996;49(6):1062–9.

[45] Doi H, Fukaya K, ichiro Oka S, Sato K, Kondoh M, Miya M. Evaluation of detection probabilities at the water-filtering and initial PCR steps in environmental DNA metabarcoding using a multispecies site occupancy model. Sci Rep [Internet]. 2019;9(1):1–8. Available from: 10.1038/s41598-019-40233-1 30837589 PMC6401178

[46] Davis GE, Kushner DJ, Mondragon JM, Mondragon JE, Lerma D, Richards D V. Kelp Forest Monitoring Handbook. C HANNEL I SLANDS N ATIONAL P ARK Channel Islands National Park. 1997;54.

[47] Lubchenco J, Gaines S, Warner R, Airamé S, Simler B. Partnership for Interdisciplinary Studies of Coastal Oceans. The science of marine reserves. PISCO. 2002;

[48] Andruszkiewicz EA, Starks HA, Chavez FP, Sassoubre LM, Block BA, Boehm AB. Biomonitoring of marine vertebrates in Monterey Bay using eDNA metabarcoding. PLoS One. 2017;12(4):1–20. doi: 10.1371/journal.pone.0176343 28441466 PMC5404852

[49] Manter DK, Weir TL, Vivanco JM. Negative effects of sample pooling on PCR-Based estimates of soil microbial richness and community structure. Appl Environ Microbiol. 2010;76(7):2086–90. doi: 10.1128/AEM.03017-09 20139317 PMC2849261

[50] Marotz C, Sharma A, Humphrey G, Gottel N, Daum C, Gilbert J, et al. Triplicate PCR reactions for 16 S rRNA gene amplicon sequencing are unnecessary. 2019;29–32. 10.2144/btn-2018-0192 PMC7030937 31124709

[51] Katija K, Dabiri JO. A viscosity-enhanced mechanism for biogenic ocean mixing. Nature. 2009;460(7255):624–6. doi: 10.1038/nature08207 19641595

[52] Katija K. Biogenic inputs to ocean mixing. J Exp Biol. 2012;215(6):1040–9. doi: 10.1242/jeb.059279 22357597

[53] Bessey C, Jarman SN, Berry O, Olsen YS, Bunce M, Simpson T, et al. Maximizing fish detection with eDNA metabarcoding. Environ DNA. 2020;2(4):493–504.

[54] Andruszkiewicz EA, Sassoubre LM, Boehm AB. Persistence of marine fish environmental DNA and the influence of sunlight. PLoS One. 2017;12(9):1–18. doi: 10.1371/journal.pone.0185043 28915253 PMC5600408

[55] Stoeckle MY, Soboleva L, Charlop-Powers Z. Aquatic environmental DNA detects seasonal fish abundance and habitat preference in an urban estuary. PLoS One. 2017;12(4):1–15. 10.1371/journal.pone.0175186 PMC5389620 28403183

[56] Stock A, Crowder LB, Halpern BS, Micheli F. Uncertainty analysis and robust areas of high and low modeled human impact on the global oceans. Conserv Biol. 2018;32(6):1368–79. doi: 10.1111/cobi.13141 29797608

[57] Evans NT, Olds BP, Renshaw MA, Turner CR, Li Y, Jerde CL, et al. Quantification of mesocosm fish and amphibian species diversity via environmental DNA metabarcoding. Mol Ecol Resour. 2016;16(1):29–41. doi: 10.1111/1755-0998.12433 26032773 PMC4744776

