# Supplementary figures and images for: Short-lived detection of an introduced vertebrate eDNA signal in a nearshore rocky reef environment
Source: PLoS One. 2021 Jun 4;16(6):e0245314. doi: 10.1371/journal.pone.0245314 (PMC8177635; doi:10.1371/journal.pone.0245314)

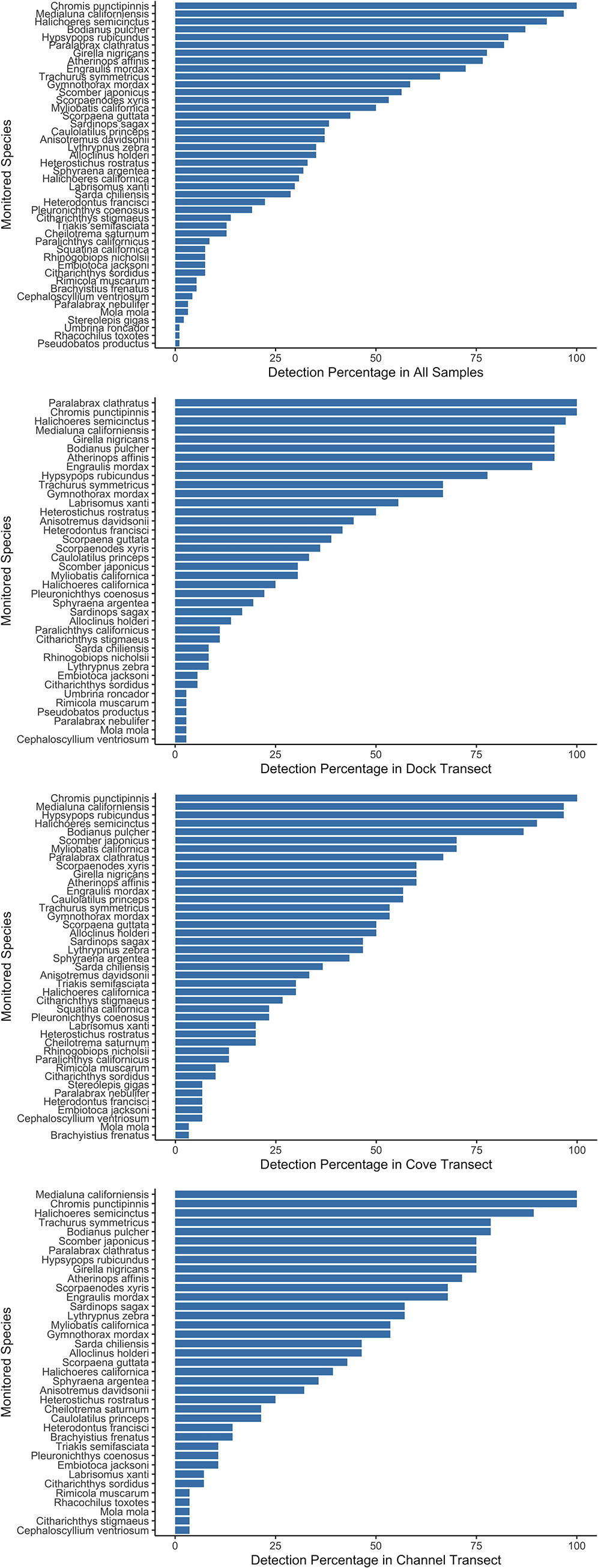

Supplement: S1 Fig — Histogram of the percentage of samples each species was detected in for all species monitored by Reef Check, Partnership for Interdisciplinary Studies of Coastal Oceans (PISCO), or National Park Service (KFM) observed for (A) all three transects, (B) only dock transect, (C) only cove transect, and (D) only channel transect. (TIF) [file pone.0245314.s001.tif]

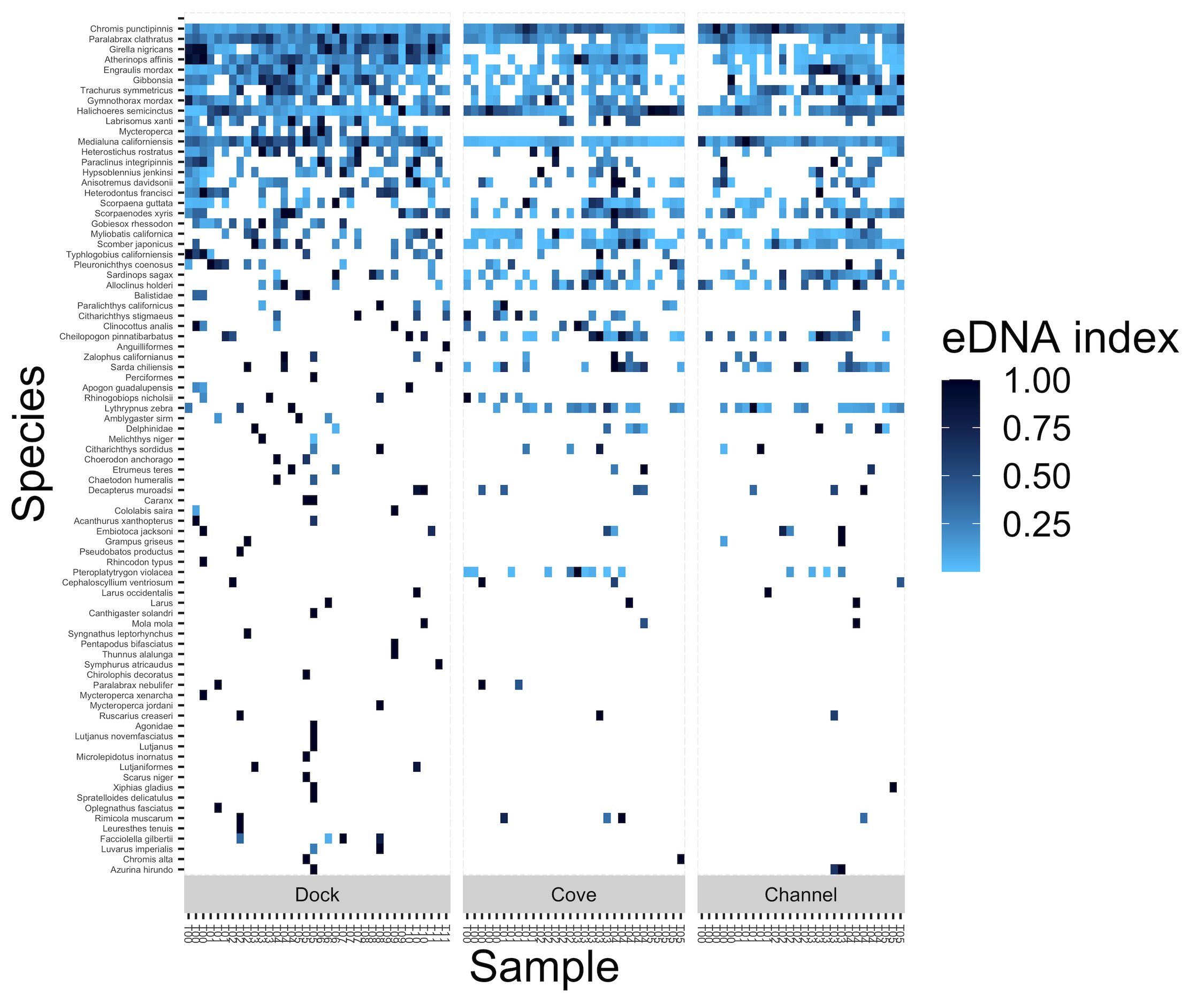

Supplement: S2 Fig — The heat map is ordered by time point then by replicate (in order of location A to E) and faceted by transect. Darker blue indicates higher eDNA index scores, interpreted as higher relative prevalence. White indicates the taxa was not detected. (TIF) [file pone.0245314.s002.tif]

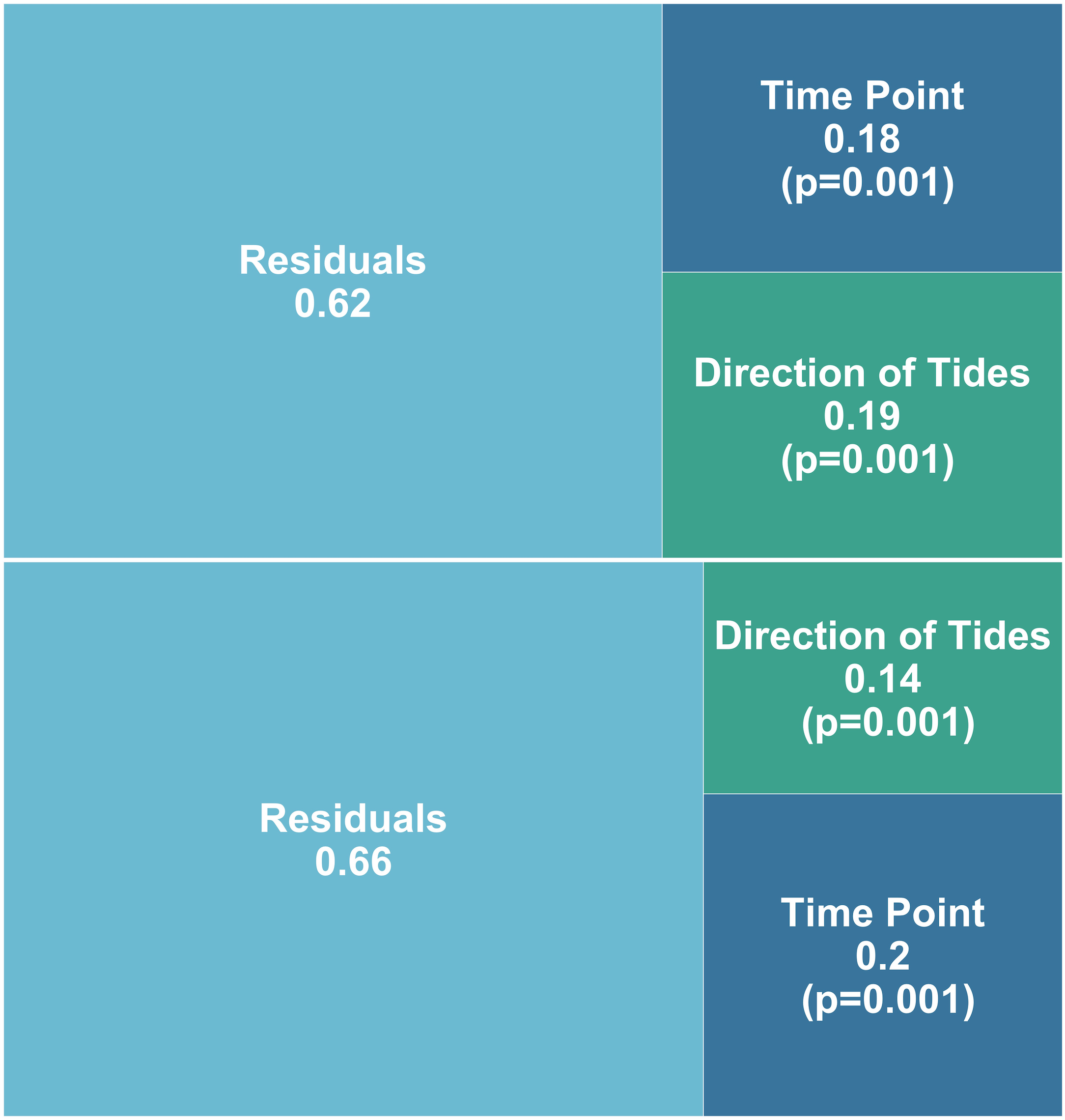

Supplement: S3 Fig — Apportioned variance plots from a PERMANOVA with Bray-Curtis dissimilarities for (A) cove transect and (B) channel transect. P-values are stated for each factor. The two processes examined are direction of tide (incoming/outgoing/peak, N = 3) and time point (0–120 hrs, N = 6). (TIF) [file pone.0245314.s003.tif]

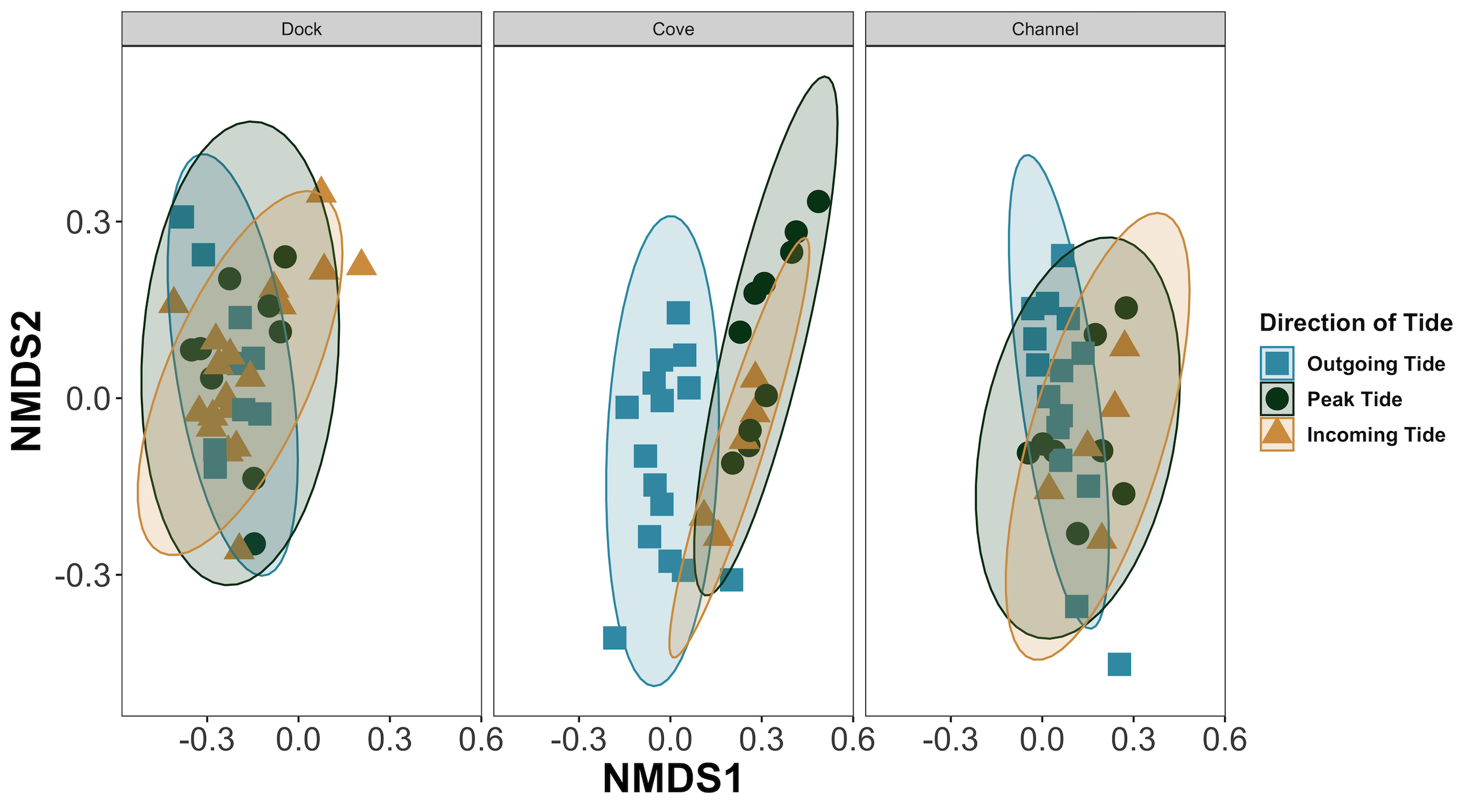

Supplement: S4 Fig — NMDS ordination plot of community assemblages using all taxa observed with Bray-Curtis dissimilarities faceted by transect. The plot is colored and filled by time point. (TIF) [file pone.0245314.s004.tif]

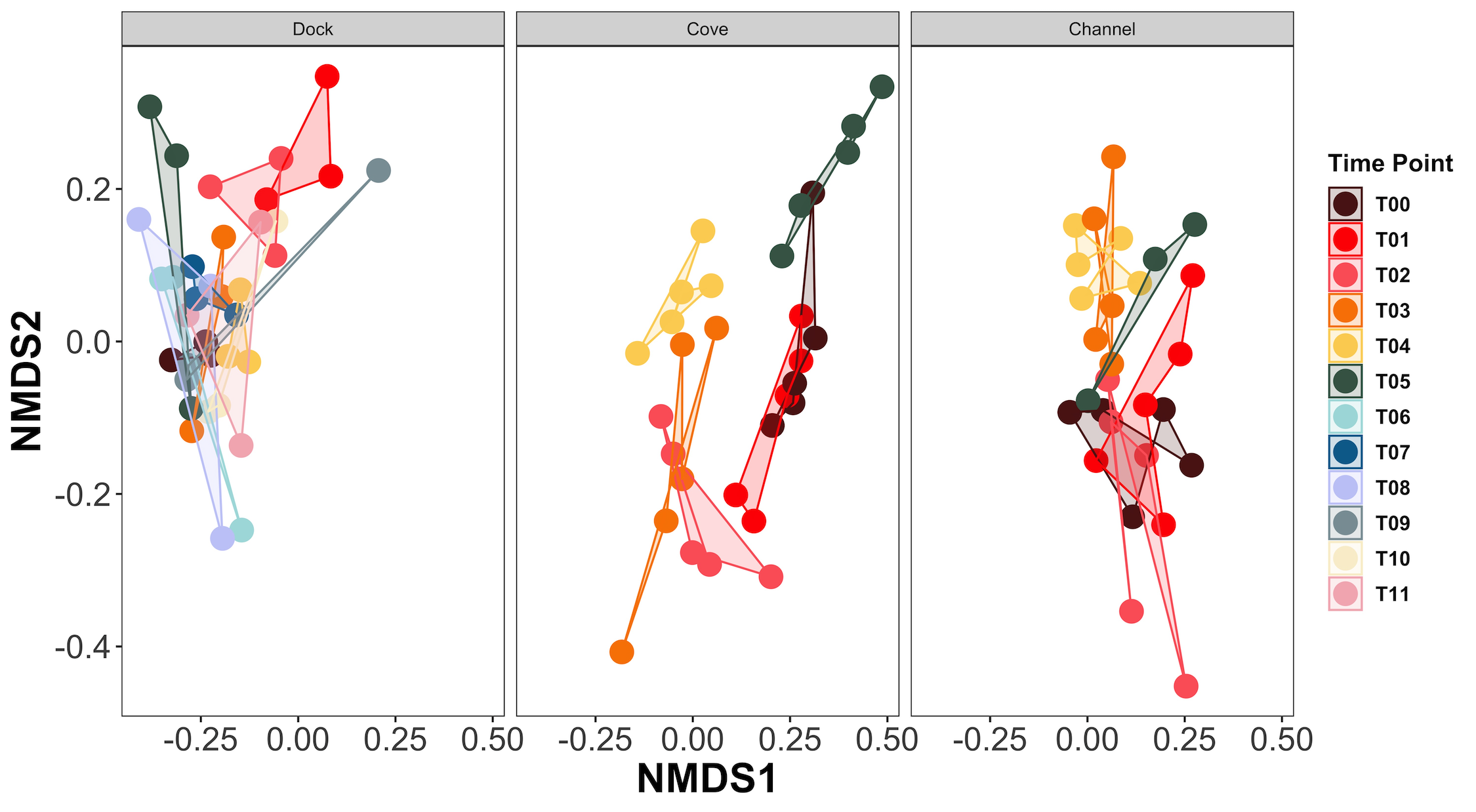

Supplement: S5 Fig — NMDS ordination plot of community assemblages using all taxa observed with Bray-Curtis dissimilarities faceted by transect. The plot is colored and filled by direction of tide (incoming/outgoing/peak). Shapes also correlate with direction of tide (incoming/outgoing/peak). (TIF) [file pone.0245314.s005.tif]

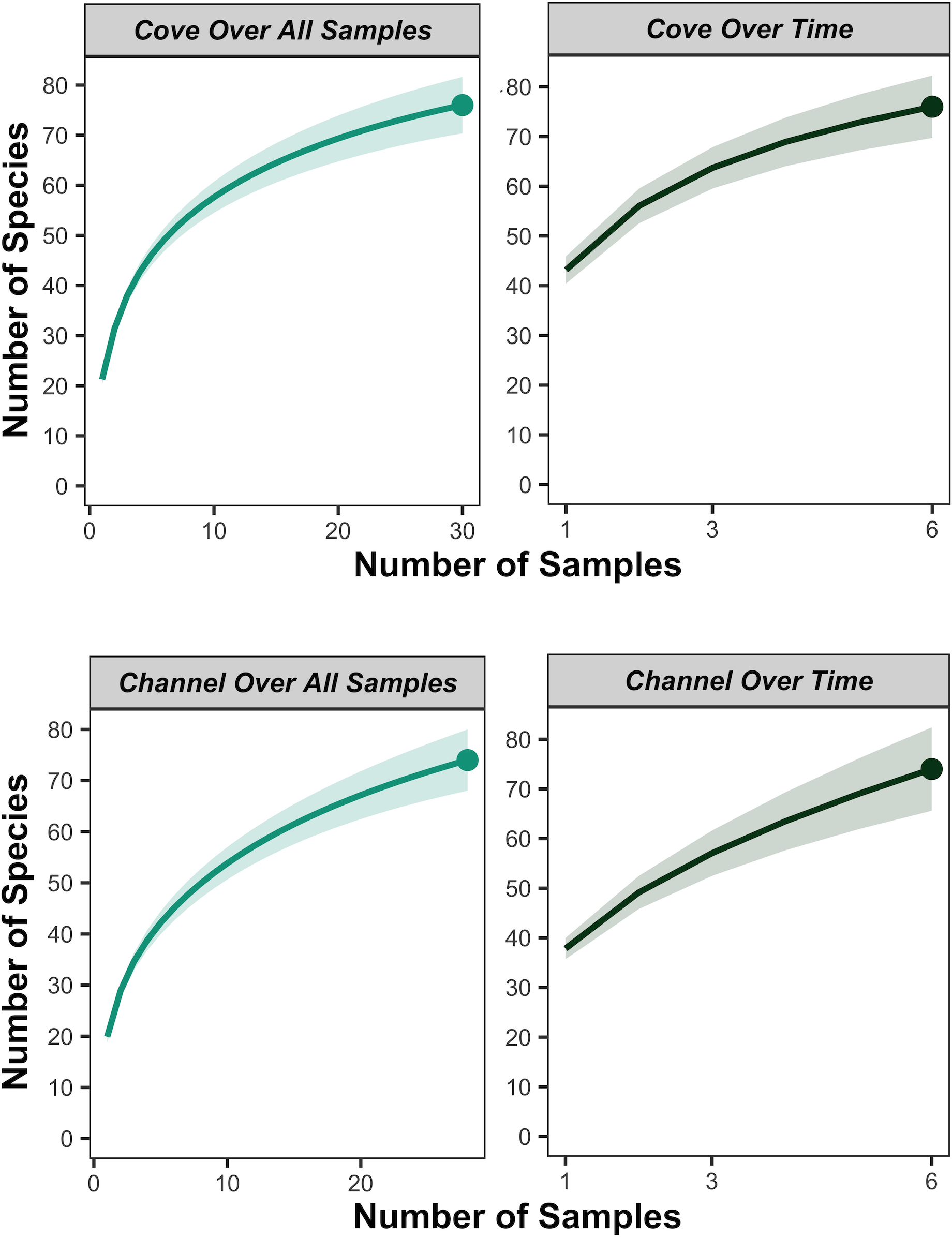

Supplement: S6 Fig — Species accumulation curves which indicate how many species on average are detected with increasing number of samples taken for (A) cove transect and for (B) channel transect. The left graph is a species accumulation curve for total marine vertebrate diversity in each replicate over all samples of each transect. The right graph is a species accumulation curve for total marine vertebrate diversity along the transect over all time points of each transect. (TIF) [file pone.0245314.s006.tif]
